# A Machine-Learning-Based Clinical Decision Model for Predicting Amputation Risk in Patients with Diabetic Foot Ulcers: Diagnostic Performance and Practical Implications

**DOI:** 10.3390/diagnostics15243142

**Published:** 2025-12-10

**Authors:** Lei Gao, Zixuan Liu, Siyang Han, Jiangning Wang

**Affiliations:** 1 Orthopedic Department, Capital Medical University Affiliated Beijing Shijitan Hospital, Beijing 100038, China; gaolei3337@bjsjth.cn (L.G.); hansiyang96@163.com (S.H.); 2 Orthopedic Department, Beijing Shijingshan Hospital, Beijing 100049, China; liuzixuan508189@163.com

**Keywords:** amputation, C-reactive protein, diabetic foot ulcer, machine learning, predictive model, support vector machine, Wagner classification

## Abstract

**Objective:** To establish a reliable machine-learning-based model for predicting the risk of lower limb amputation in patients with diabetic foot ulcers and to provide quantitative evidence for clinical decision-making and individualized prevention strategies. **Methods:** This retrospective study analyzed data from 149 hospitalized diabetic foot ulcer patients treated at Beijing Shijitan Hospital between January 2019 and December 2022. Patients were divided into amputation and non-amputation groups according to clinical outcomes. Candidate predictors—including infection biomarkers, vascular parameters, and nutritional indices—were first screened using the least absolute shrinkage and selection operator algorithm. Subsequently, a support vector machine model was trained and internally validated via five-fold cross-validation to estimate amputation risk. Model performance was evaluated by discrimination, calibration, and clinical utility analysis. **Results:** Among all enrolled variables, C-reactive protein and Wagner grade were identified as independent predictors of amputation (*p* < 0.05). The optimized support vector machine model achieved excellent discrimination, with an area under the Receiver Operating Characteristic curve of 0.89, and demonstrated a moderate level of calibration (Hosmer–Lemeshow χ^2^ = 19.614, *p* = 0.012). Decision curve analysis showed a net clinical benefit of 0.351 when the threshold probability was set at 0.30. The model correctly classified 82.4% of cases in internal validation, confirming its predictive robustness and potential for clinical application. **Conclusions:** C-reactive protein and Wagner grade are key determinants of amputation risk in diabetic foot ulcer patients. The support vector machine-based prediction model exhibits strong accuracy, clinical interpretability, and personalized interventions.

## 1. Introduction

Diabetic foot (DF) disease, which includes ulcers, infections, and gangrene of the feet, is one of the leading causes of disability worldwide. Due to the high disability rate and expensive treatment cost of diabetic foot, doctors and patients all hope to forecast the prognosis in time and give early intervention. With the development of artificial intelligence technology, more and more methods are used in the diagnosis and prognosis prediction of chronic diseases. Machine learning, a type of artificial intelligence, has excellent predictive effects with a certain accuracy [1]. The results of diabetic foot are affected by many factors, so it is necessary for the machine learning to reasonably predict the relationship between input variables and output variables and to correct and tolerate faults [2]. The prevalence of type 2 diabetes (T2DM), a complex and inordinate metabolic disease, is becoming greater [3]. DFU is a major global challenge for older people. It is one of the leading causes of disability worldwide [4]. Life expectancy is affected due to complications such as infections and amputations. People with diabetes have a 25 percent lifetime risk of developing foot ulcers, and 14 to 24 percent require severe gangrene or minor lower limb amputation [5]. Therefore, early and accurate prediction of amputation has guiding significance for improving the quality of life and survival rate of DFU patients. It is necessary to establish a predictive model to predict the risk of amputation in DFU patients. The decision integration of clinical diagnosis and treatment with long-term prognosis was attempted to improve the quality of prognosis, reduce the amputation rate of DFU patients, and reduce treatment costs.

The classification of the severity of diabetic foot infection has been the focus of international diabetic foot scholars. The Meggitt–Wagner classification is the most widely used rating system for evaluating the development of diabetic foot [6]. The higher the Wagner rating, the greater the likelihood of amputation, and the lower the cure and recovery rate [7]. The University of Texas diabetic wound classification is an improvement on the Wagner scale, which associated wound depth with ischemic infection [8]. WiFi classification has been improved on the basis of the earlier classification, integrating ulcer area, ischemia index and foot infection degree [9]. It can assess the severity of diabetic foot patients from a multi-dimensional perspective, which is one of the most widely used classification systems at present. Alb also reflects nutritional status to some extent, and relevant studies have shown that Alb level is negatively correlated with the severity of diabetic foot. Therefore, Alb is an outstanding indicator for predicting the risk of amputation of diabetic foot ulcers [10]. These methods are common tools for clinical diagnosis and long-term prediction of diabetic foot patients, but these are not the gold standard for clinical diagnosis. Hence, the prediction of diabetic foot amputation will be a comprehensive consideration of blood supply, wound, nutrition, infection, and other factors, not simply relying on subjective judgment but rather on scientific analysis. In addition, the current classification system does not take into account the basic information of the patient, such as age, gender, and medical history, which affects the accuracy and scientific nature of clinical decision-making to a certain extent.

Early identification and targeted prevention of diabetic foot are of great significance for improving patient prognosis and reducing medical burden [11]. Risk prediction model refers to estimating the probability or risk of the existence (diagnostic model) or future occurrence (prognostic model) of a specific disease or condition through mathematical formulas [12,13]. Previous study used traditional statistical methods (e.g., multiple logistic regression analysis and COX proportional risk model) to predict the risk of amputation in DFU patients [14]. However, due to the diversity and unpredictability of the influencing factors, the prediction range of these methods is limited [15,16]. In recent years, with the continuous improvement of the understanding of medical big data and in-depth research on statistical methods, machine learning (ML) algorithms can predict the occurrence and prognosis of diseases [17,18,19]. This offers a novel perspective for clinical decision-making and improves predictive efficiency. Support vector machines (SVMs) are supervised learning algorithms that can be applied to both regression tasks and binary classification problems. It reduces the error caused by empirical classification and increases the margin, also known as the maximum margin classifier [20]. At present, SVM has been widely used in the medical field, but it is mainly used in the prognosis assessment of cancer patients, and rarely used in the field of survival analysis, especially in the field of chronic diseases [21,22,23].

The primary objective of this study intends to develop a machine learning model based on SVM algorithm that can predict the amputation rate of diabetic foot ulcers. In addition, this study tries to integrate clinical diagnosis and treatment with long-term prognosis decision-making, providing scientific guidance for clinical decision-making and nursing work of diabetic foot ulcer, improving the quality of prognosis and reducing the rate of amputation.

The remainder of this manuscript is structured as follows. In Section 2, the study design, participant selection criteria, data collection procedures, and the statistical and machine-learning methodologies—including preprocessing steps, LASSO-based feature selection, and classifier construction—are described in detail.

Section 3 reports the main results, including the selected predictors, performance metrics of the machine learning models, confusion matrices, calibration analyses, and decision curve evaluation. Section 4 discusses the implications of the findings, their relevance within the existing literature, the methodological contributions of the work, and the study’s limitations. Finally, Section 5 presents the conclusions and summarizes the potential clinical applications of the proposed prediction model.

## 2. Methods

### 2.1. Inclusion and Exclusion Criteria for Subjects

This research randomly selected diabetic foot infection patients admitted to the Diabetic Foot Centre Department of Beijing Shijitan Hospital Affiliated to Capital Medical University, from January 2019 to December 2022, as the study subjects to carry out this retrospective cohort study.

The study included patients who met the following criteria:

(a) Admitted to hospital with a diagnosis that met DFU’s clinical diagnostic criteria;
(b) Diabetic foot patients above Wagner level 1;
(c) Routine laboratory tests and auxiliary examinations have been completed after admission;
(d) Surgical treatment has been performed during the visit;
(e) The number of hospitalizations of the patient within the investigation range ≤2 times;
(f) This study protocol was known to the patient, and the patient himself was informed and consented.

Exclusion criteria included:

(a) Patients with other infectious diseases;
(b) Patients with malignant tumors;
(c) Patients younger than 18 years;
(d) Patients transferred to other healthcare facilities during treatment.

The prognosis of the patients was split into two types according to the surgical method: (a) amputation group; (b) non-amputation group. According to the plan of amputation, amputation can be divided into minor amputation, which is considered to be below the ankle amputation, and severe amputation, which is above the ankle amputation.

### 2.2. Subject Inclusion Index

A formal a priori sample size calculation was not performed because this study used a retrospective dataset. A total of 149 patients with diabetic foot ulcers (DFU) were included in this study. For each patient, it collected comprehensive demographic, clinical, and laboratory information, including age, gender, number of admissions, date of hospitalization, history of hyperlipidemia, wound location, Wagner classification, and final clinical outcome (amputation or non-amputation). Laboratory indicators consisted of C-reactive protein (CRP, mg/L), procalcitonin (PCT, ng/mL), white blood cell count (10^9^/L), albumin (g/L), and the degree of arteriosclerosis of the lower extremity assessed by Doppler ultrasonography.

The entire cohort (*n* = 149) was divided into two outcome categories: amputation group (*n* = 68) and non-amputation group (*n* = 81). For model development, the dataset was further split using five-fold stratified cross-validation into a training set of 120 patients (amputation = 54 and non-amputation = 66) and a validation set of 29 patients (amputation = 14, non-amputation = 15).

### 2.3. Data Preprocessing

In order to ensure the accuracy and scientificity of input variables and reduce systematic errors, the prediction model was first fitted. The Lasso (Least Absolute Shrinkage and Selection Operator) algorithm can initially screen the predictors and obtain a model with strong performance and simplicity. It takes the lambda value corresponding to the cross-validation error within 1 standard deviation of the minimum error as the optimal penalty coefficient of the model. Then, multivariate logistic regression analysis was carried out on the variables selected by LASSO regression using backward likelihood method to determine the final predictors and construct the nomogram model.

### 2.4. Statistical Analyses

Descriptive statistical analysis of the data was performed for each of the two groups separately. In addition, continuous variables were expressed as mean ± standard deviation. For continuous variables, normality was assessed using the Kolmogorov–Smirnov test [24]. Variables that did not follow a normal distribution were compared between groups using the Wilcoxon rank-sum test [25]. Categorical variables were compared using the Chi-square test [26]. In addition, categorical variables are expressed as counts (*n*) and percentages (%). A *p*-value < 0.05 was considered statistically significant. All the above calculations were performed using SPSS 21.0.

### 2.5. Model Development

Machine-learning algorithms were employed to construct amputation-risk prediction models. After applying the LASSO regression for variable selection, all continuous predictors were z-score standardized, and the dataset was split using five-fold stratified cross-validation to ensure balanced training and validation subsets. Three complementary classifiers—support vector machine (SVM), linear discriminant analysis (LDA), and k-nearest neighbors (KNN)—were developed in MATLAB2019A. These algorithms were selected to provide linear (LDA), non-parametric instance-based (KNN), and non-linear margin-based (SVM) decision boundaries appropriate for small-to-moderate clinical datasets.

The SVM classifier was implemented using the fitcsvm function with a radial-basis-function kernel (KernelFunction = ‘rbf’). The box-constraint parameter was set to C = 1.0, the kernel scale was determined automatically from the data (KernelScale = ‘auto’), and predictors were standardized during training (Standardize = true). Empirical class priors were applied. The LDA classifier was constructed using the fitcdiscr function with a linear discriminant function (DiscrimType = ‘linear’). No additional regularization was introduced (γ = 0). Empirical class priors were used, and all predictors were standardized before model fitting. The KNN classifier was trained using the fitcknn function with k = 5 neighbors (NumNeighbors = 5), Euclidean distance (Distance = ‘euclidean’), and squared-inverse distance weighting (DistanceWeight = ‘squaredinverse’). Predictors were standardized prior to training (Table S1).

After model construction, prediction performance was assessed using confusion matrices, discrimination metrics, calibration plots, and decision curve analysis to identify the optimal predictive model.

### 2.6. Model Assessment and Validation

Model performance was assessed through a comprehensive validation framework designed to determine predictive accuracy, calibration, and clinical utility. Three primary discrimination metrics—the area under the receiver operating characteristic curve (AUC), sensitivity, and specificity—were calculated for each classifier to quantify model accuracy in distinguishing amputation from non-amputation outcomes. Calibration curves were generated to evaluate the agreement between predicted and observed risks, thereby assessing the model’s goodness-of-fit. In addition, decision curve analysis (DCA) was conducted to quantify the net clinical benefit across a range of threshold probabilities and to determine whether the predictive model improves clinical decision-making over default strategies such as treating all or no patients.

After LASSO regression identified the most informative predictors, five-fold stratified cross-validation was applied to evaluate model stability and generalizability. For each fold, the selected predictors were re-estimated using the training subset and then evaluated in the held-out subset, ensuring that both variable selection and model assessment were conducted without information leakage. This validation strategy reflects how the model would perform when applied to new and unseen clinical cases.

Although LASSO regression was used as the initial variable-selection method, the final predictors included in the nomogram were derived from the subsequent multivariable logistic regression using the backward likelihood approach. This step ensured that all variables contributing statistically or clinically meaningful information were retained. As recommended by TRIPOD (Transparent Reporting of a multivariable prediction model for Individual Prognosis Or Diagnosis) guidelines, the nomogram was constructed from the final regression model rather than strictly from the LASSO-selected variables, thereby enhancing clinical interpretability and allowing a more comprehensive representation of individualized risk.

### 2.7. Flowchart of the Study Design and Model Construction

**Figure 1:**
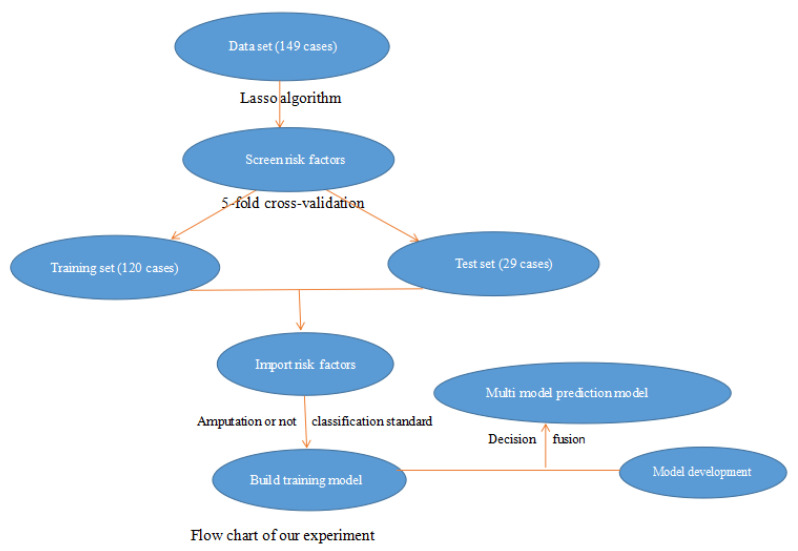
Flowchart of the study design and model construction.

The workflow for developing the machine-learning-based amputation-risk prediction model (Figure 1). The process included patient selection according to inclusion and exclusion criteria, data collection from 149 diabetic-foot-ulcer (DFU) cases, variable screening using the least absolute shrinkage and selection operator (LASSO) regression, five-fold cross-validation to divide the dataset into training and validation subsets, and construction of multiple machine learning models (LDA, K-nearest neighbor, and SVM). Model performance was assessed through discrimination, calibration, and decision curve analysis to determine the optimal predictive model.

## 3. Results

### 3.1. Statistical Test Result

The overall analysis of infection indicators between the amputation and non-amputation groups is summarized in Table 1 and Table 2.

In the drawing, *T* test was used to analyze continuous variables, and Chi-square test was used to analyze categorical variables. Among them, variables CRP, PCT, and WBC of the training set and test set did not conform to normality, so a U test was used for analysis. According to the analysis results, in the training set, there were statistically significant differences between CRP, PCT, WBC, and Wagner_rating in terms of generalized amputation scores (*p* < 0.05, see overall data status).

Multivariate analysis results (Table 3) showed that CRP, Wagner_rating3, and Wagner_rating4 were independent predictors of generalized amputation (all *p* < 0.05). The degree of vascular sclerosis and vascular occlusion in one or both lower limbs did not affect the prognosis of amputation.

### 3.2. Determine Input Variable

**Figure 2:**
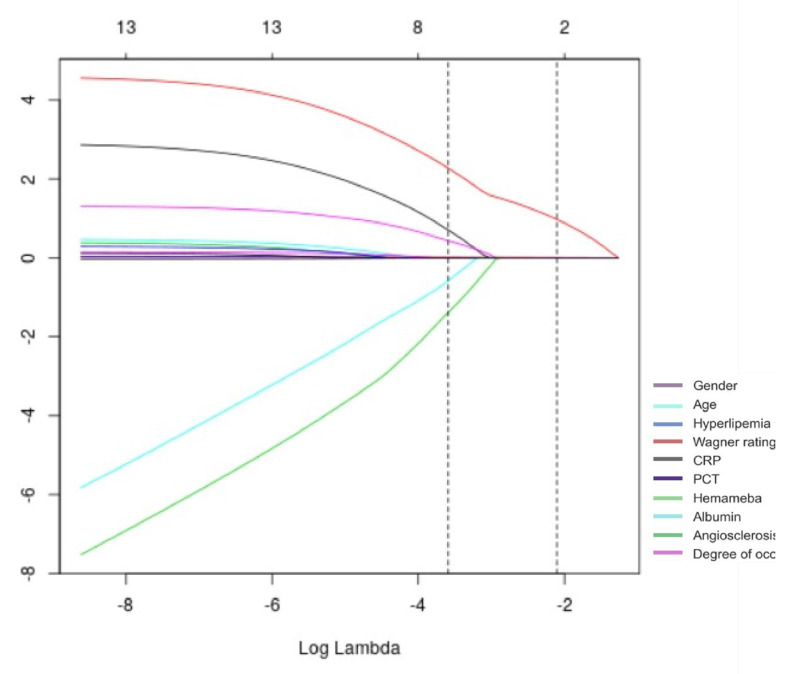
LASSO shrinkage coefficient path diagram.

The shrinkage path of the LASSO regression coefficients is illustrated in Figure 2. The figure shows the noose coefficient contours for 10 texture features. The ten texture features refer to quantitative gray-level co-occurrence matrix (GLCM) parameters, including contrast, correlation, energy, homogeneity, entropy, dissimilarity, variance, cluster shade, cluster prominence, and angular second moment. The horizontal coordinate is the penalty coefficient, and the vertical coordinate is the gene coefficient. A coefficient profile is plotted against the log(λ) sequence, and the vertical line is plotted at selected values that are cross-validated by a factor of 10, where the optimal λ yields four non-zero coefficients. This is basically consistent with the results of statistical analysis. LASSO regression was used to screen the relevant risk factors, and then this study calculated the corresponding regression coefficients to improve the robustness of the model [27].

**Figure 3:**
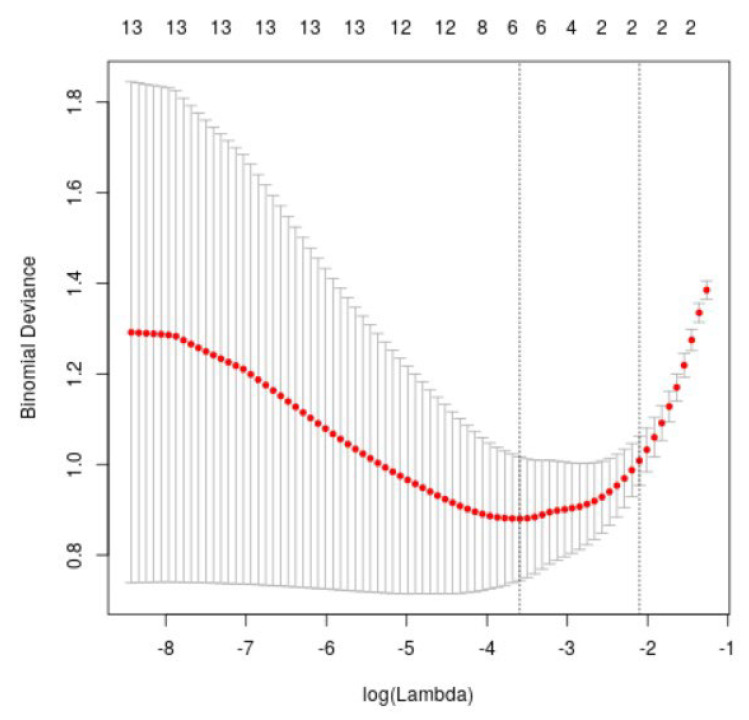
LASSO regression analysis cross-validation curve. The two vertical dotted lines represent the optimal penalty parameters selected by 10-fold cross-validation. The left dotted line corresponds to λ_min, the value of λ that yields the minimum binomial deviance. The right dotted line corresponds to λ_1se, the largest λ within one standard error of the minimum deviance, which provides a more parsimonious model with fewer non-zero coefficients. The red curve (red points connected by a line) shows the average binomial deviance obtained from 10-fold cross-validation for each log(λ) value. Each red point corresponds to one candidate λ tested during regularization. The vertical grey bars represent the standard error of the cross-validation deviance, indicating the variability of the model’s performance across different folds. Narrower grey bars indicate more stable estimates, whereas wider bars reflect higher uncertainty.

The ten-fold cross-validation curve used to determine the optimal λ value is presented in Figure 3. The figure shows the cross-verification curve of LASSO regression analysis, where the horizontal coordinate is the penalty coefficient and the vertical coordinate represents the cross-verification error. The smaller the value of the vertical axis, the better the LASSO fitting effect. Using the minimum criterion and one standard error of the minimum criterion (1-se criterion), the vertical dotted line is drawn at the optimal value. After 10-fold cross-verification, the λ value is 0.0276, and the log(λ) is 0.122 (1-SE criterion). At the same time, the upper horizontal coordinate corresponding to this point is the number of variables that can be used for analysis.

### 3.3. Model Performance

**Figure 4:**
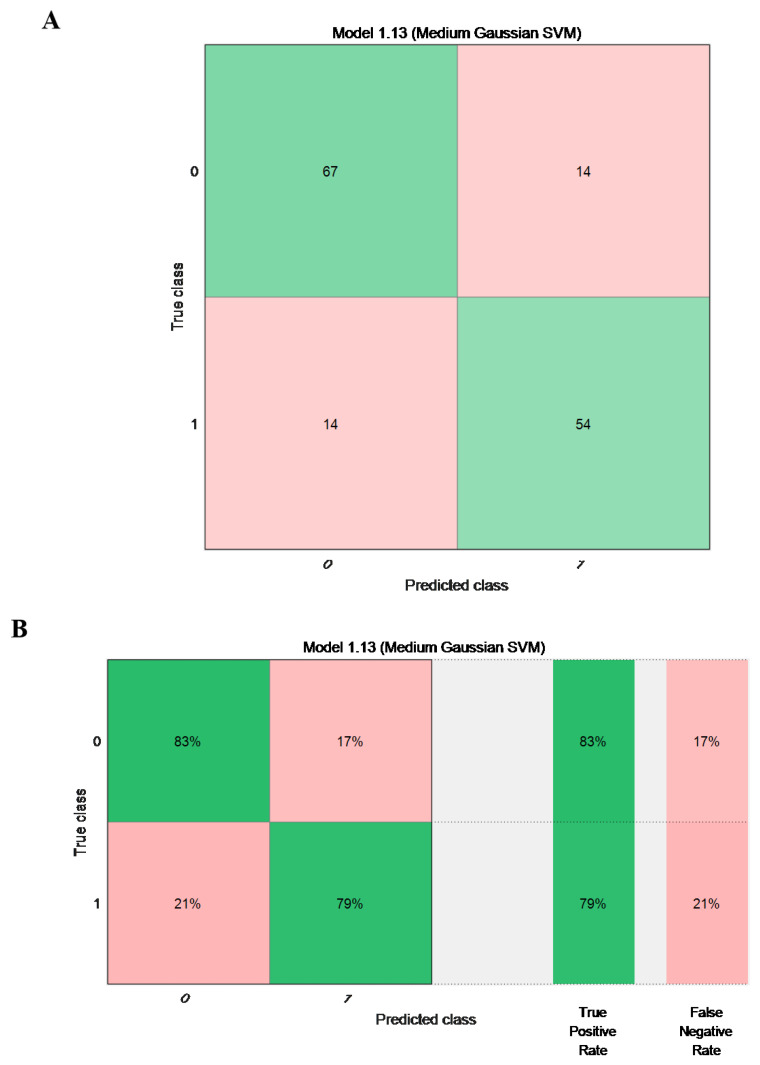
Confusion matrix drawn from prediction model. (**A**) presents the raw confusion matrix showing the absolute numbers of true positives, true negatives, false positives, and false negatives. (**B**) shows the same results expressed as percentages to visualize the proportional classification performance for each class (amputation vs. non-amputation). Green cells represent correctly classified samples (true positives and true negatives), whereas pink cells indicate misclassified samples (false positives and false negatives).

The confusion matrix generated from the SVM-based prediction model is displayed in Figure 4. This is a confusion matrix, plotted by a predictive model built from the data set. The formulation of confusion matrix makes the prediction result more intuitive and convenient for clinical workers to make a judgment and decision [28]. As shown in the figure, the predicted results are consistent with the actual amputation results in the green part, while in the pink part, the two are completely opposite. The SVM model achieved an accuracy of 82.4% based on the internal performance evaluation. This accuracy was consistent with the results reflected by the confusion matrix, which showed a similar classification performance. Therefore, the SVM model demonstrated reliable discriminative ability for amputation prediction.

Among the three classifiers (LDA, KNN, and SVM), SVM achieved the highest overall performance, with the largest AUC (0.89), strongest sensitivity-specificity balance, and the highest F1-score (0.812), supporting its selection as the final prediction model (Table 4).

This study tried to use SVM method to build a prediction model, run the prediction model according to the customized function, calculate the accuracy of the model, and select the function with the highest accuracy as the modeling decision tree. The prediction accuracy of the model reached 82.4%, which is consistent with the overall performance of the classifiers. This indicates that the model provides meaningful guidance for clinical decision-making.

### 3.4. Model Evaluation

**Figure 5:**
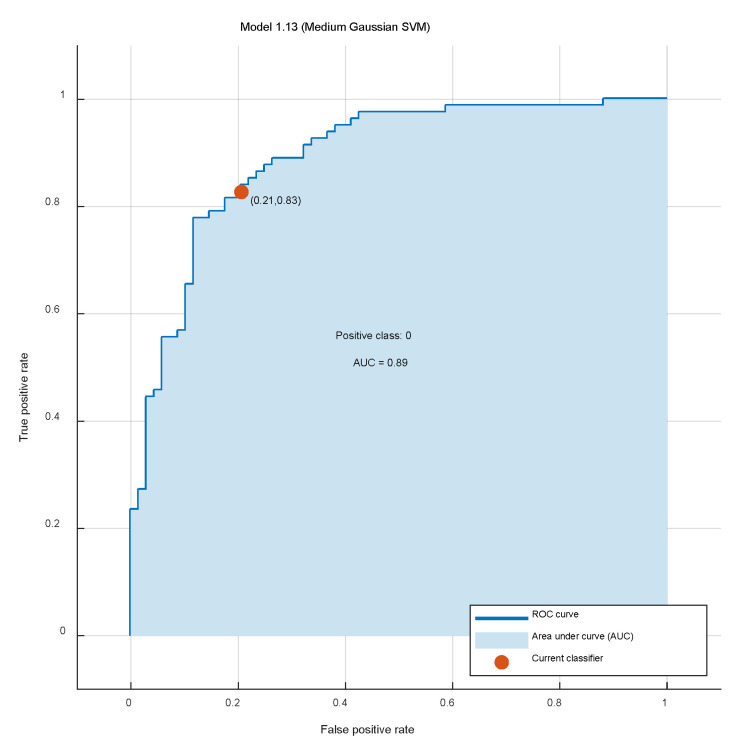
Accuracy and calibration performance of the prediction model.

The receiver operating characteristic (ROC) curve demonstrating model accuracy and calibration performance is shown in Figure 5. The ROC is plotted based on whether generalized amputation or not, where the area under the receiver operating characteristic curve (AUC) is 0.89. The closer the curve area is to 1, the higher the accuracy of the proof model. The maximum approximate entry index (Max (sensitivity + specificity − 1)) under the ROC curve is supreme when the tangent point is 0.21, corresponding to 0.83.

**Figure 6:**
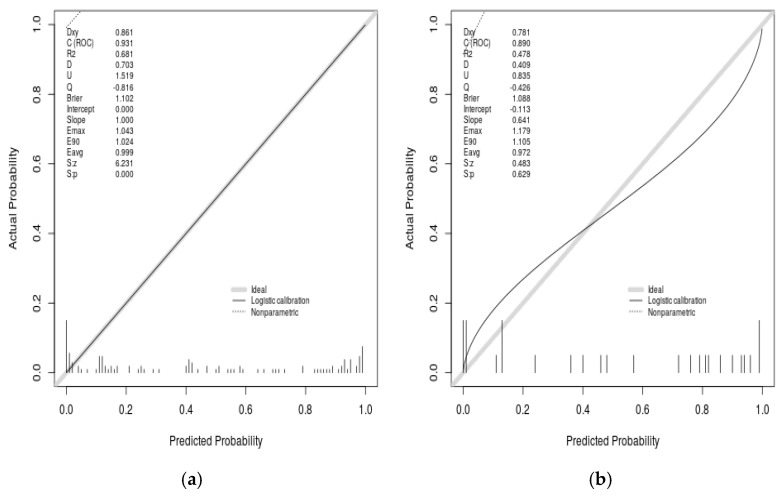
Calibration curve of amputation prediction model. (**a**) Model calibration curve on the training set; (**b**) Model calibration curve on the test set.

The calibration curves for the training and validation datasets are provided in Figure 6a,b to assess the model’s agreement between predicted and observed outcomes. The calibration ability of the model was 19.614 (*p* = 0.012) through the Hosmer–Lemeshow test, suggesting some deviation from ideal agreement between predicted and observed risk, particularly at the extreme ends of the predicted probability range. Figure 6a is the calibration curve of the model on the training set; Figure 6b is the calibration curve of the model on the test set. Calibration curves are drawn based on the agreement between the observed and predicted risk of amputation and the actual results. The *Y*-axis represents the actual amputation outcome, and the *X*-axis represents the predicted risk of amputation. The gray diagonal line represents an ideal perfect model, while the solid black line represents the performance of the model, and the closer the two lines are, the better the fit.

### 3.5. Multimodal Analysis

**Figure 7:**
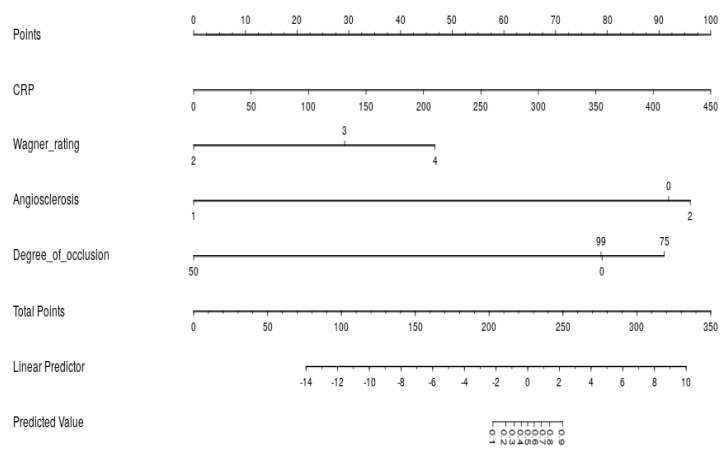
A nomogram of the amputation prediction model.

A visual representation of the amputation prediction model integrating all independent predictors is presented as a nomogram in Figure 7. The nomogram of the prediction model based on whether generalized amputation occurs or not is shown in Figure 4, integrating all independent predictors.

**Figure 8:**
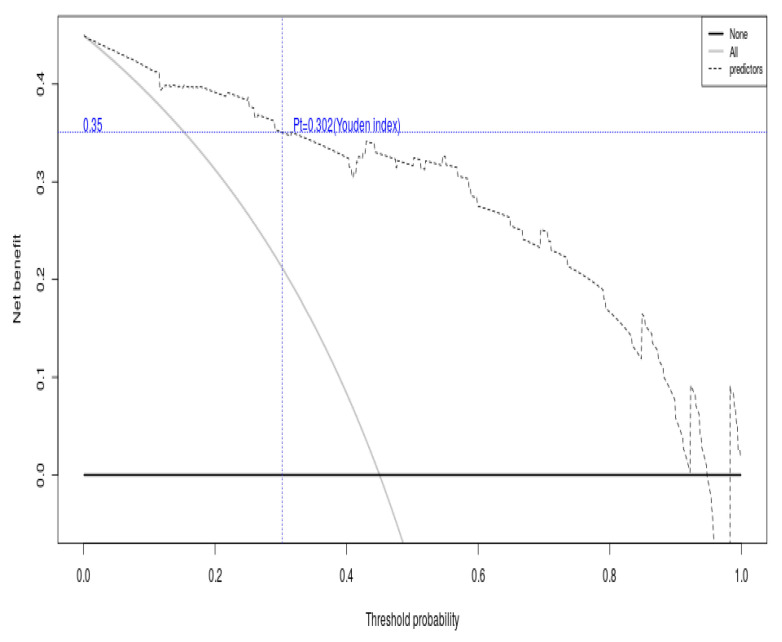
Decision analysis curve of diabetic foot amputation.

The clinical decision curve illustrating the net benefit across threshold probabilities is shown in Figure 8. The graph decision curve analysis shows the net benefit of the model to the patient as the threshold selection changes. When the threshold value is selected as 0.302 derived from the Jorden index, the model is able to generate a net gain of 0.351.

## 4. Discussion

In this study, an amputation prediction model was developed that incorporates 10 baseline features to predict the probability of amputation in DFU patients. The AUC and calibration capability of the prediction model were 0.89 and 19.614, respectively. Regarding calibration, the Hosmer–Lemeshow *p*-value of 0.012 indicates that the model exhibited only moderate calibration, with some over- or under-prediction at the extremes of risk. This reflects the limitations inherent in using a single-center retrospective dataset and highlights the need for larger datasets and external validation to refine the calibration performance. At the same time, according to the decision analysis curve, when the threshold is 0.302, the net benefit is 0.351. This shows that the prediction model has strong clinical application prospects, and that the predicted probability of the model is in reliable agreement with the actual probability. Numerous studies have shown that the more severe the infection, the higher the amputation rate in DFU patients [29,30]. In the prediction model of amputation prognosis of DFU patients, a machine learning model with infection severity, lower limb blood supply, and systemic nutrition as input variables was established. The analysis showed that in the patient cases we collected, the prognosis of patients with amputation was affected by multiple factors, such as severe infection, lower limb vascular occlusion, poor nutritional status, etc., which was basically consistent with clinical experience and related studies [31,32]. In addition, it is noteworthy that hyperlipidemia was associated with an increased risk of amputation. These findings are meaningful and consistent with established clinical observations.

The Wagner classification system is widely used in clinical practice to assess the severity of foot ulcers in diabetic patients. It categorizes foot ulcers into six grades [33]. The classification of wounds is based on depth, extent, and the degree of infection, all of which require careful clinical assessment. This additional information helps to improve the accuracy of the model in predicting the risk of amputation in DFU patients. A prediction model for amputation risk and further improve the DFU classification system needs to be built from multiple aspects [34,35,36,37]. Overall, incorporating the Wagner classification system into the predictive model allows for a more comprehensive assessment of the risk of amputation in DFU patients. It enhances the predictive power of the model and provides valuable clinical information for personalized treatment and management of diabetic foot disease [37].

In addition, simple linear models (COX regression, multiple Logistic regression, and COX proportional risk model) have certain limitations in assessing the prognosis of DFU patients due to the diversity and unpredictability of influencing factors. Boyko et al. [38] first carried out a prospective cohort study on diabetic patients in 2006, applied Cox proportional risk model to screen independent influencing factors, and finally formed a scoring system model. The AUC of this model was 0.81, indicating strong differentiation. But the study was not externally verified. In 2019, British scholar Heald et al. [14] analyzed diabetic patients through retrospective cohort study and Logistic multiple regression, finally included five risk factors and built a calculation equation for the risk probability of diabetic foot based on the regression coefficients of each factor. The model AUC was 0.65. Although the model has certain clinical practicability, the accuracy rate is still low. Tomita et al. [39] conducted a case–control study, and the AUC of the constructed model was 0.865. The emergence of artificial intelligence provides new ideas and methods for diabetes risk prediction. In 2021, Peng et al. [40] constructed a model to predict the risk of diabetic foot amputation and adopted the nomogram to visually compare the risk weights of each risk factor. Nonetheless, the sample size and input variables of this experiment are insufficient. The model constructed is not verified. Deng et al. [19] used XGBoost algorithm and COX regression to evaluate the impact of hyperglycemic crisis and other risk factors on the mortality of DFU patients. The model’s AUC is 0.680. A prospective study by Lv et al. [41] established a DFU risk model based on risk factors and presented it in the form of the nomogram and web calculator. The AUC of its model was 0.741. Therefore, machine learning algorithms adopt a multivariate, non-parametric approach that can use non-normal distributions and strongly correlated data to build robust models and identify complex patterns [42,43]. Compared with the statistical methods in previous relevant studies, the prediction model this study constructed includes more relevant factors, has higher accuracy, and more intuitive prediction results.

The integration of model prediction with clinical decision-making represents a key feature of this study. This study intends to construct a database of diabetic foot amputation patients, study the real world of diabetic foot amputation patients, and validate predictive models to guide clinical decision-making. Obviously, whether the predictive model based on retrospective data analysis can be applied to clinical practice needs to be further verified. Therefore, it hopes to collect the information of newly included diabetic foot patients, compare the model prediction results, optimize the internal algorithm of the model, further screen the relevant risk factors, and understand the distribution of diabetic foot characteristics in the current survey population. Based on the relevant risk factors it has screened, it should proceed with the multi-disciplinary combined diagnosis and treatment of diabetic foot patients from the four aspects of nutrition, blood circulation, wound surface, and infection. It is essential for the early prevention, timely intervention, and scientific individualized treatment of DFU [44,45]. Several previous studies have attempted to use machine-learning approaches to predict amputation risk in patients with diabetic foot ulcers. Liu et al. [46] developed a comparative machine learning model to predict major amputation. Their primary goal, however, was to benchmark multiple algorithms, whereas the present study aims to build a clinically interpretable and deployable tool that integrates LASSO-based variable selection, multivariable modeling, calibration, and decision curve analysis. Moreover, this model predicts the risk of both minor and major amputations, capturing a broader and more clinically relevant spectrum of outcomes.

However, several limitations should be acknowledged. Although this study uses SVM algorithms to build models, it still lacks sufficient clinical validation and cohort studies. It is true that machine learning models primarily establish mapping relationships between input variables and predicted outcomes, rather than capturing direct causal relationships. Identifying causal relationships in complex medical conditions such as foot ulcers is challenging. Machine learning models can provide insights into the associations between patient characteristics and predicted outcomes, but further investigation and studies are needed to establish causality. It is essential to consider the timing and effectiveness of treatment interventions when interpreting the predictions made by the model. The fluctuation of the infection index of a single patient’s second admission is influenced by the treatment measures. At the same time, it can not ignore that the decision of diabetic foot amputation surgery is affected by many aspects, such as the patient’s economic status, wound status, and the subjective judgment of clinicians, and it is difficult to predict whether amputation is possible only through observational indicators. The aim is to develop a model that differs from the existing DFU taxonomy system and can be optimized and refined on the basis of the Wagner classification. In this way, highly accurate and clinically practical models were constructed to predict amputation risk in patients with DFU, with an additional effort to link predictive modeling to both clinical care and long-term prognosis. Although the individual techniques applied in this study—such as LASSO regression, logistic modeling, and machine-learning classifiers—are well-established, the methodological contribution of this study lies in integrating these components into a clinically interpretable and decision-oriented prediction framework. By combining LASSO-based dimensionality reduction with multivariable modeling and comparative machine-learning classifiers and further validating performance through calibration and decision curve analyses, this study presents a hybrid approach that differs from earlier DFU models. Moreover, the construction of a nomogram from the final regression model bridges the gap between algorithmic performance and bedside usability, offering a transparent, individualized risk estimation tool that adheres to TRIPOD guidelines and supports clinical decision-making. Another important limitation is the lack of external validation. Because this study was based on a single-center retrospective cohort, only internal validation could be performed. Although cross-validation and regularization techniques reduce overfitting, the generalizability of the model to different populations, time periods, and clinical environments remains uncertain. External validation—either through a temporal split within the same institution or through independent multi-center cohorts—is necessary to confirm the robustness and real-world applicability of the prediction model. Future work will focus on validating the model using datasets from additional centers and across different clinical settings.

## 5. Conclusions

This study built an intelligent model which can be used to forecast the risk of inpatient amputation in DFU patients and analyze the real world of newly enrolled diabetic foot patients. The experimental results show that the machine learning model not only has accurate predictive power but also provides new ideas for the formulation of personalized treatment plans for patients.

## Figures and Tables

**Table 1:** Overall analysis of infection indicators in diabetic foot amputation patients (Age, Gender, CRP, PCT, WBC).

		Age		Gender		CRP		PCT		WBC	
		Mean (SD)	Median [Min, Max]	Male	Female	Mean (SD)	Median [Min, Max]	Mean (SD)	Median [Min, Max]	Mean (SD)	Median [Min, Max]
Training Dataset	non-amputation (*n* = 66)	64.8 (11.9)	65.0 [34.0, 91.0]	47 (71.2%)	19 (28.8%)	36.0 (51.9)	14.0 [0.400, 292]	0.249 (0.704)	0.05 [0.01, 4.98]	9.09 (3.80)	7.83 [2.00, 26.4]
	amputation (*n* = 54)	63.7 (11.5)	64.0 [39.0, 88.0]	41 (75.9%)	13 (24.1%)	137 (93.5)	122 [1.15, 411]	0.859 (1.99)	0.175 [0.02, 11.1]	12.0 (5.84)	11.4 [3.96, 31.1]
	*p*-values	0.601		0.561		<0.001		<0.001		0.0042	
Validation Dataset	non-amputation (*n* = 15)	67.2 (14.1)	67.0 [42.0, 88.0]	9 (60.0%)	6 (40%)	48.2 (72.7)	18.2 [1.23, 265]	0.438 (0.934)	0.08 [0.03, 3.59]	10.2 (5.88)	8.67 [4.93, 24.9]
	Amputation (*n* = 14)	66.4 (16.6)	65.5 [39.0, 96.0]	8 (57.1%)	6 (42.9%)	125 (88.7)	106 [2.46, 332]	0.311(0.729)	0.045 [0.02, 2.76]	12.2 (5.60)	9.85 [5.14, 22.8]
	*p*-values	0.884		1		0.006		0.154		0.183	

**Table 2:** Overall analysis of infection indicators in diabetic foot amputation patients (Albumin, Hyperlipemia, Wagner Rating, Angiosclerosis, Degree of Occlusion).

		Albumin		Hyperlipemia		Wagner Rating			Angiosclerosis			Degree of Occlusion			
		Mean (SD)	Median [Min, Max]	No	Yes	Grade 2	Grade 3	Grade 4	Mild	Moderate	Severe	0	50%	75%	99%
Training Dataset	non-amputation (*n* = 66)	34.6 (5.95)	34.7 [17.1, 46.4]	50 (75.8%)	16 (24.2%)	29 (43.9%)	23 (34.8%)	14 (21.2%)	4 (6.1%)	2 (3.0%)	60 (90.9%)	33 (50.0%)	2 (3.0%)	4 (6.1%)	27 (40.9%)
	amputation (*n* = 54)	33.7 (4.68)	33.7 [20.5, 45.1]	37 (68.5%)	17 (31.5%)	1 (1.9%)	11 (20.4%)	42 (77.8%)	4 (7.4%)	0 (0%)	50 (92.6%)	18 (33.3%)	0 (0%)	9 (16.7%)	27 (50.0%)
	*p*-values	0.385		0.377		<0.001			0.647			0.0609			
Validation Dataset	non-amputation (*n* = 15)	33.4 (5.84)	34.7 [23.0, 43.0]	11 (73.3%)	4 (26.7%)	5 (33.3%)	7 (46.7%)	3 (20.0%)	0 (0%)	1 (6.7%)	14 (93.3%)	7 (46.7%)	0 (0%)	0 (0%)	8 (53.3%)
	Amputation (*n* = 14)	32.7 (6.14)	32.8 [22.7, 44.4]	9 (64.3%)	5 (35.7%)	0 (0%)	2 (14.3%)	12 (85.7%)	0 (0%)	0 (0%)	14 (100%)	7 (50.0%)	0 (0%)	3 (21.4%)	4 (28.6%)
	*p*-values	0.756		0.7		0.00103			1			0.16			

**Table 3:** Multivariate analysis of infection index in diabetic foot amputation patients.

	β	Odds Ratio (95% CI)	*p*
(Intercept)	−5.1546		
Angiosclerosis moderate	−17.8435	<0.001	0.994
Angiosclerosis severe	0.3903	1.477[0.119, 18.306]	0.761
CRP	0.0207	1.021[1.010,1.032]	<0.001
Degree of occlusion 50	−16.5541	<0.001	0.994
Degree of occlusion 75	1.1200	3.065[0.367, 25.597]	0.301
Degree of occlusion 99	−0.0199	0.980[0.277, 3.473]	0.975
Wagner rating3	2.7226	15.220[1.535, 150.867]	0.020
Wagner rating4	4.3520	77.630[8.152, 739.265]	<0.001

**Table 4:** Performance comparison of the three machine learning models.

Model	AUC	Sensitivity	Specificity	Accuracy	Precision	F1-Score
LDA	0.81	0.723	0.777	0.795	0.745	0.734
KNN	0.78	0.722	0.851	0.819	0.808	0.763
SVM	0.89	0.796	0.865	0.824	0.828	0.812

## Data Availability

The original contributions presented in this study are included in the article. Further inquiries can be directed to the corresponding author(s).

## Supplementary Materials

The following supporting information can be downloaded at: https://www.mdpi.com/article/10.3390/diagnostics15243142/s1, Table S1. Machine-learning model parameters used in the study.
