# Supplementary material for: A Machine-Learning-Based Clinical Decision Model for Predicting Amputation Risk in Patients with Diabetic Foot Ulcers: Diagnostic Performance and Practical Implications
Source: Diagnostics (Basel). 2025 Dec 10;15(24):3142. doi: 10.3390/diagnostics15243142 (PMC12731953; doi:10.3390/diagnostics15243142)
Supplement: Supplementary file 1 [file diagnostics-15-03142-s001.zip › diagnostics-3984334-supplementary.pdf]

Table S1. Machine-learning model parameters used in the study

Model	Software Function	Key Parameters
SVM	fitcsvm	Kernel = RBF ('rbf'), BoxConstraint = 1.0, KernelScale = 'auto', Standardize = true, ClassPrior = empirical
LDA	fitcdiscr	DiscrimType = 'linear', Regularization γ = 0, ClassPrior = empirical, Predictors standardized
KNN	fitcknn	NumNeighbors = 5, Distance = 'euclidean', DistanceWeight = 'squaredinverse', Standardize = true
